# One Core to Root them All: A Core Bacterial Community in Closely Related but Geographically Distant *Mammillaria* Species from Contrasting Environments

**DOI:** 10.1007/s00248-026-02854-8

**Published:** 2026-08-06

**Authors:** Joel Colchado-López, Cristian R. Cervantes, Andrea Jiménez-Marín, Nguyen Esmeralda López-Lozano, Roberto Garibay-Orijel, Bruno M. Chávez-Vergara, Ulises Rosas

**Affiliations:** 1 Jardín Botánico, Instituto de Biología, Universidad Nacional Autónoma de México, Circuito Zona Deportiva S/N, Ciudad Universitaria, Coyoacán, Mexico City, 04510 Mexico; 2 Posgrado en Ciencias Biológicas, Unidad de Posgrado, Universidad Nacional Autónoma de México, Circuito de Posgrados S/N, Ciudad Universitaria, Coyoacán, Mexico City, 04510 Mexico; 3 Unidad de Síntesis en Sistemática y Evolución, Instituto de Biología, Universidad Nacional Autónoma de México, Circuito Zona Deportiva S/N, Ciudad Universitaria, Coyoacán, Mexico City, 04510 Mexico; 4 Instituto de Biología, Pabellón Nacional de la Biodiversidad, Universidad Nacional Autónoma de México, Circuito Cultural S/N, Ciudad Universitaria, Coyoacán, Mexico City, 04510 Mexico; 5 División de Biología Molecular, SECIHTI - Institute for Scientific and Technological Research of San Luis Potosí (IPICYT) MX, 78216 San Luis Potosí, San Luis Potosí Mexico; 6 Departamento de Botánica, Instituto de Biología, Universidad Nacional Autónoma de México, Circuito Zona Deportiva S/N, Ciudad Universitaria, Coyoacán, Mexico City, 04510 Mexico; 7 Instituto de Geología, Universidad Nacional Autónoma de México, Circuito Interior S/N, Ciudad Universitaria, Coyoacán, Mexico City, 04510 Mexico; 8 Laboratorio Nacional de Geoquímica y Mineralogía, Universidad Nacional Autónoma de México, Circuito Interior S/N, Ciudad Universitaria, Coyoacán, Mexico City, 04510 Mexico

**Keywords:** Arid environments, Cacti, Holobiont, Rhizosheath, Rhizosphere, Xerophytes

## Abstract

Understanding the core microbiome has potential applications on biomonitoring and conservation. We explore whether closely related xerophytes from the *Mammillaria* haageana species complex (MHSC), a recently diverged group (ca. 2 mya) that occupy geographically distant and contrasting environments, share a bacterial core microbiome (BCM) that could help them to thrive through an aridity gradient. We sampled the rhizosheath and the surrounding bulk soil, performed 16S rRNA (v3-v4 region) metabarcoding, and analyzed soil properties, bacterial diversity and functional predictions, to compare between soil compartments and describe the BCM associated to the MHSC rhizosheath. We observed that bacterial communities differed among bulk soils but MHSC species display a trend to select for the same bacteria. Despite environmental differences, we found that all four species shared a BCM composed of 17 bacterial taxa, assigned to genera *Dactylosporangium*, *Microvirga*, and *Rubrobacter*; as well as 14 predicted functional processes. This BCM could enhance rhizosheath nutrient acquisition and stress responses, thus helping the plant-bacteria holobiont to thrive in arid environments and help us understand the rapid evolution and diversification of the MHSC across an aridity gradient

**Supplementary Information:**

The online version contains supplementary material available at https://doi.org/10.1007/s00248-026-02854-8.

## Introduction

The core microbiome describes the microbial entities that persist across hosts in a set of populations. This concept helps us understand microbial community assembly and establishment in the host tissues, with applications on environmental biomonitoring, assessing wildlife health and species conservation [1–2]. Thus, the core microbiome may under stable conditions facilitate the establishment of the microbial community on the “holobiont”, a coevolutionary entity formed by a healthy microbial community and its host [3].

Traditionally, the core microbiome is defined as the set of microorganisms that are consistently found across a population of hosts or environments. This ‘common core’ is restricted to highly abundant microorganisms that occur in most of the individuals. Nevertheless, this definition faces major limitations, such as the lack of a strict universal core when environmental or host behaviors are highly variable. New definitions of the core microbiome concept are summarized by Risely [4]. One is the ‘functional core’ concept [5], where hosts select for microbes that provide key ecological services for its host. This functional core can be identified by direct measurements (e.g. metatranscriptomic), or inferred as predicted functional potentials using pipelines such as PICRUST2 [6].

One of the most studied systems for understanding host-microbe relations are the plant roots. Both organisms interact though the soil region known as the rhizosphere, on which root organic matter attract microbes that provide the roots with benefits such as Nitrogen (N) fixation, Phosphorous (P) acquisition, bioweathering to augment soil availability, biomining and chelation [7], and stress alleviation [8]. One function critical for arid climates is the ‘rhizosheath’, a specialized rhizosphere modification.

The rhizosheath is a trait found in xerophytes that prevents root dehydration and allows for an increased tolerance of nutrient deficiency by attaching soil macroaggregates to the hair roots and promoting biofilm production to fulfill the holobiont physiological needs. This is such an efficient process that current research has proposed this mechanism as a means of sustainable agriculture under the current climatic crisis [9]. However, it is important to mention that the rhizosheath structure depends on soil properties, and is also responsive to the genetic background of its host [10].

Among the more extensively researched rhizosheath communities are those from the xerophytic family Cactaceae. Bacterial communities have been characterized in distantly related cacti such as *Cereus jamacaru* [11], *Mammillaria carnea*, *Opuntia pilifera* and *Stenocereus stellatus* [12], *Myrtillocactus geometrizans* and *O. robusta* [13], and *O. ficus-indica* [14]. Bacterial communities in cacti differ from the bulk soil, but also across different host tissues. In addition, these communities respond to aridity, aiding host survival across environmental gradients. Nevertheless, it is not fully understood how the core microbiome varies within recently divergent species that thrive on different soil conditions across an aridity gradient.

*Mammillaria haageana* species complex (MHSC) comprises six species of recent divergence (ca. 2.3 mya according to Cervantes et al. [15]). They occupy contrasting environments across an arid-to-sub-humid spectrum, including the Reserva del Pedregal de San Angel (Mexico City), where *Mammillaria san-angelensis* is found and serves as a model species for cacti [16].

Given this recent diversification, some bacteria may have co-evolved with MHSC, enabling colonization of new environments. In this scenario, their bacterial core microbiome (BCM) might consist of shared taxa across the species complex (common core), or alternatively, by a recruitment strategy on recurrent biological services (functional core). Although these concepts are distinct notions, they have not been considered simultaneously when describing the BCM of closely related species. Therefore, here we describe the common and functional BCM of four species from the MHSC. We aim to disentangle (i) if the filtering patterns of each species share common bacterial elements, (ii) if we can find a set of common taxonomic units (common core), and (iii) if these taxonomic units comply with predicted functional potentials that could be positively selected by the MHSC species. Finally, we discuss how the characterization of the BCM is relevant to understanding the recent diversification of the MHSC species complex and the importance of BCM in xerophyte evolution for survival in arid environments.

## Methods

### Study Sites and Biological Sampling

**Fig. 1:**
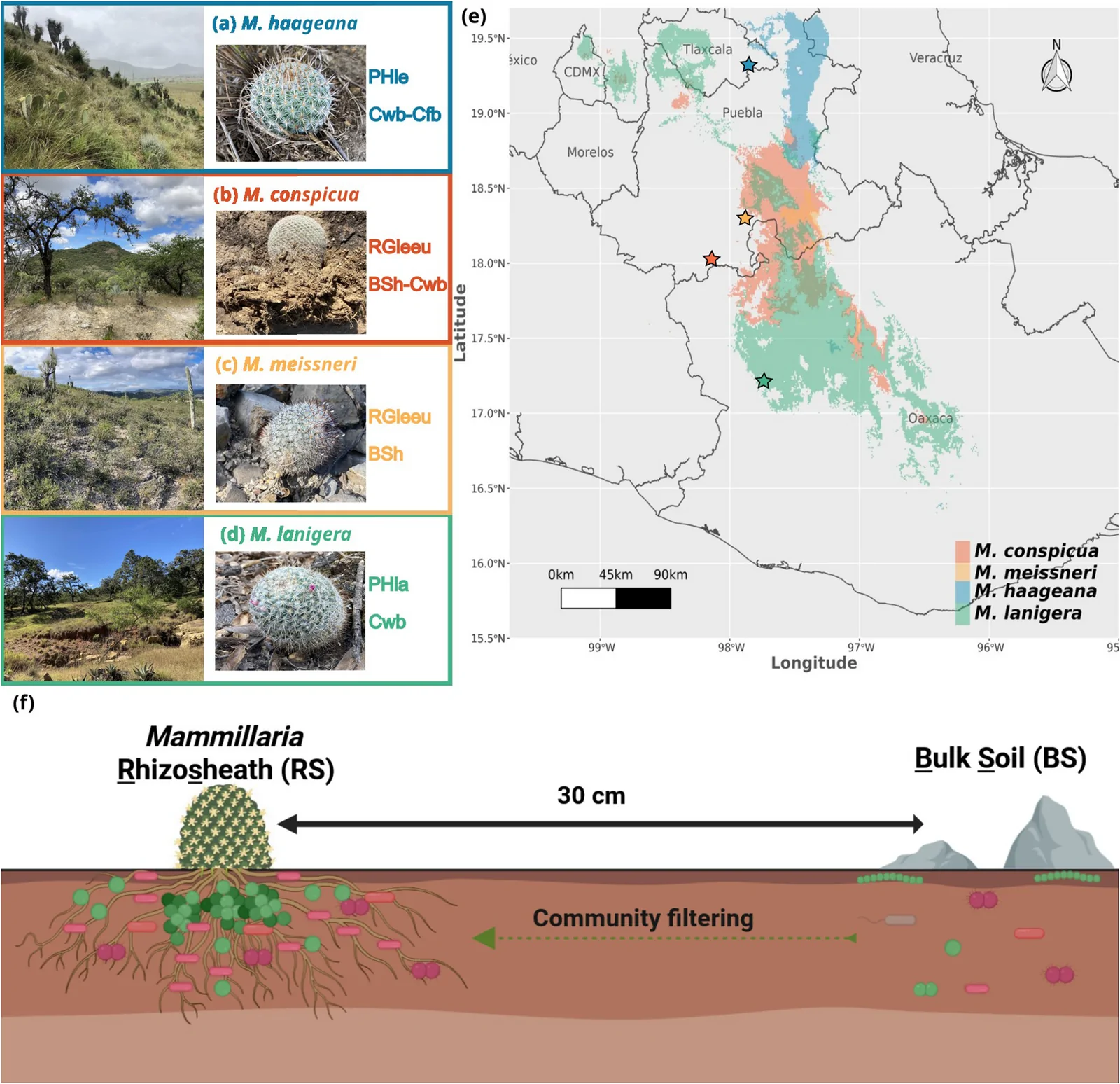
The *Mammillaria haageana* species complex (MHSC) and soil compartments. Four localities (all in Mexico), each one associated to a different species of the MHSC, were sampled: (**a**) *M. haageana* from Perote, (**b**) *M. conspicua* from Chazumba, (**c**) *M. meissneri* from Zapotitlán, and (**d**) *M. lanigera* from Nochixtlan. Inside each box, the top code (e.g. PHle) refers to soil type according to WRB soil classification, while the bottom code (e.g. Cfb) corresponds to weather type according to Köppen. Geographical distribution is shown on the map (**e**), with stars indicating the sampled localities. (**f**) Schematic definition of the two sampled compartments – *r*hizo*s*heath (RS) and *b*ulk *s*oil (BS) – highlighting our hypothesized filtering process whereby the MHSC species RS recruit similar bacterial communities, taxonomically or functionally, from distinct BS communities

We sampled four MHSC species across Central and Southern Mexico (Fig. 1a-d), distributed across distinct biogeographic provinces (Transmexican Volcanic Belt, Balsas Basin, and Sierra Madre del Sur) following the scheme by Morrone et al. [17]. Each locality harbored a single species, and the sites are distributed along a North-to-South gradient spanning nearly 250 km (Fig. 1e). Soils were classified according to the World Reference Base (WRB) classification system [18]. The sampled species and brief descriptions of their environment are as follows: (i) *Mammillaria haageana* Pfeiff. from a xerophytic shrubland on leptosol phaeozems (PHle) on hill slopes in Perote, Veracruz (Fig. 1a). The climate was subtropical highland under a rain shadow (Cwb-Cfb), with recurrent sporadic drizzle. (ii) *M. conspicua* J.A.Purpus from a dry seasonal forest in Chazumba, Puebla (Fig. 1b), under hot semi-arid to subtropical highland climates (BSh-Cwb), rarely found associated with another plant and occasionally found near slopes with eutric regosols (RGleeu) formed by landslides. (iii) *M. meissneri* C.Ehrenb. from the Cuicatlán-Tehuacán Desert Biosphere Reserve at Zapotitlán, Puebla (Fig. 1c); this species was often found as groups of two or three individuals and rock nursing was widespread. The climate was hot semi-arid (BSh) with RGleeu soils. (iv) *M. lanigera* Repp. from the most humid environment sampled; these plants were associated with a *Quercus acutifolia* forest in Nochixtlán, Oaxaca that barely stood over the ecocline with a dry seasonal forest (Fig. 1d). The climate was Cwb; this location was the only site with clayey haplic phaeozems soils (PHha) and a thick leaf litter layer.

Sampling occurred at the end of the rainy season. At each location, we chose nine sampling points separated by at least 10 m and not situated on the same slope. From each point we collected nine 1 kg *b*ulk *s*oil (BS) samples (Fig. 1f) and sealed them in 26.8 × 27.3 cm Ziploc plastic bags and stored at 3° C, to evaluate eight soil biogeochemical and physical properties. Furthermore, we chose five out of those nine points to sample the *Mammillaria* *r*hizo*s*heath (RS), defined as the soil tightly adhering to the roots. To minimize plant influence on the soil community, BS was sampled 30 cm from RS and any visible vegetation, at a maximum depth of 10 cm. Each RS sample was collected from a single adult individual. Plants were removed from the ground, roots shaken to remove loosely attached soil, and the remaining tightly adhering soil was stored as RS in a 15 mL Falcon tube. We also took subsamples from the corresponding BS samples and stored them in 15 mL Falcon Tubes. This resulted in a total of 40 samples for metabarcoding (5 RSs and 5 BSs from each of the 4 locations). All samples were immediately flash-frozen in liquid nitrogen and stored at -70 °C until environmental DNA (eDNA) extraction and 16 S rRNA metabarcoding.

### eDNA Extraction and Metabarcoding

We isolated eDNA from 250 mg of soil using the DNeasy PowerSoil Kit (QIAGEN) following the manufacturer’s protocol. Library preparation and sequencing were performed by BGI (Shenzhen, China) on an MGI DNBseq-G400 PE300 platform. The V3-V4 hypervariable region (465 bp) of the 16S rRNA gene was amplified with primers 341F (5’-CCTACGGGNGGCWGCAG-3’) and 806R (5’-GACTACHVGGGTATCTAATCC-3’). These primers are widely used for microbiome research due to their high coverage of Domain Bacteria [19, 20]. Each of the 40 sequenced samples aimed at yielding 100,000 paired-end reads. Paired-end demultiplexed reads were quality filtered and trimmed using Qiime2 (version 2024.2 [21]). Plug-in Cutadapt (version 5.6 [22]) was trained on primers 341F/806R, allowing a 0.2 error rate, wildcards and a minimum read length of 250 bp. Trimmed sequences were then denoised and merged using DADA2 (version 1.26.0 [23]), with a maximum number of expected errors of 12 for both forwards and reverse reads, and no truncation allowed (‘--p-trunc-len-f 0’, ‘--p-trunc-len-r 0’). Chimeric sequences were removed using the pooled method (‘--p-chimera-method ‘pooled’’) with a minimum parent abundance of 4. This yielded 3,293,447 pre-rarefied paired-end reads from all 40 samples, with a minimum of 35,704 reads per sample (Supplementary Information Table S1).

Amplicon Sequence Variants (ASVs) were taxonomically classified with the SILVA (version 138 [24] trained for the V3-V4 region with primers 341 F/806R. Annotation was conducted using ‘qiime feature-classifier classify-sklearn’ with a confidence cutoff (--p-confidence) of 0.8 and automatic detection of read orientation (--p-read-orientation auto). ASVs identified as Archaea, Chloroplast, Mitochondria, Eukaryote or of unknown affinity were removed using the ‘qiime taxa filter-table’ feature. Then, singletons were removed using ‘qiime feature-table filter-seqs’. Finally, the feature table was rarefied without replacement to 35,704 sequences per sample, the minimum observed across all samples, to standardize sampling for downstream comparative analyses.

### Biostatistics, Diversity Analyses and Functional Predictions

Data was exported from Qiime2 for further analyses in R (version 4.4.3 [25]). Diversity indices were calculated and visualized using packages ‘phyloseq’ (version 1.50.0 [26]), ‘vegan’ (version 2.6.10 [27]), and ‘microbiomeutilities’ (version 1.0.17 [28]). ‘ggvenn’ (version 0.1.10 [29]) was used for Venn diagrams to visualize the shared elements between the MHSC species. Analyses in R used Kruskal-Wallis functions from the ‘agricolae’ (1.3.7 [30]) package to test for statistical differences in relative taxa proportion across samples. Customized heatmaps were created with ‘ComplexHeatmap’ (version 2.22.0 [31]).

Functional potentials were predicted using PICRUSt2 (version 2.5.2 [6]), which infers metabolic capacities based on the relationship between ASVs and a set of microbial reference genomes. The resulting predictions were mapped to MetaCyc (pathways) and Kyoto Encyclopedia of Genes and Genomes (KEGG Orthology, also known as KO terms) databases. KO terms abundances were normalized with ALDEx2 (version 1.38.0 [32]), then visualized using ‘ggplot2’ (version 4.0.2 [33]).

Full details of the pipeline are available at https://github.com/jcolchadolopez/Core_MHSC.

### Soil Biochemical and Physical Analyses

In parallel with the metabarcoding, we also collected nine bulk soil samples per location, resulting in a total of 36 samples. Samples were sieved with a 2 mm wire-mesh and visible roots, leaves or animal matter and rocks (approximately over 1 cm diameter) were manually removed prior further analyses.

5 g soil subsamples were diluted in 30 mL Mili-Q water to measure (i) pH and (ii) electrical conductivity using an Orion Versa StarPro pH-meter (Thermo Scientific). Additionally, 10 g soil subsamples were used to estimate (iii) fresh and dry soil weight; soils were dried in an oven at 40 °C for 4 days. (iv) C mineralization (CO_2_) was measured using 50 g of soil moisturized below field capacity and incubated at 25 °C following the protocol described by Araújo et al. [34]; CO_2_ content was sampled at 3 and 7 days after incubation. Enzymatic activity essays were conducted for the corresponding enzymes using 3 g of soil, following protocols by Jasso-Flores et al. [35], for (v) chitinase (N cycle), (vi) phosphomonoesterase (P cycle), (vii) polyphenol oxidase and (viii) β-glycosidase (C cycle) after incubating during 4 h.

## Results

### Relative Abundancess of Major Bacterial Groups Vary Between Localities and Soil Compartments

We obtained 12,553 ASVs from all 40 samples. ASVs annotated as Unknown were 8.58% at phylum level (the lowest proportion), increasing to 51.84% at the genus level. The most abundant phyla were Acidobacteriota, Actinobacteriota (a.k.a. Actinomycetota) and Proteobacteoriota (a.k.a. Pseudomonadota). These groups differed significantly across BS samples according to Kruskal-Wallis tests with Holm correction (Acidobacteriota: H_(3)_ = 10.520, *p* = 0.015*; Actinobacteriota: H_(3)_ = 13.594, *p* = 0.004**; Proteobacteriota: H_(3)_ = 15.789, *p* = 0.001**; respectively). When comparing between soil compartments, Acidobacteriota did not vary significantly (H_(3)_ = 5.309, *p* = 0.151^ns^). Given the high number of taxa at each infra-phylum rank, we chose to rank them from highest to lowest relative abundance and calculate the abundance elbow point to display the most abundant taxa at order (24 orders) and family (29 families) level for an improved readability, and less arbitrary choice of what is presented.

**Fig. 2:**
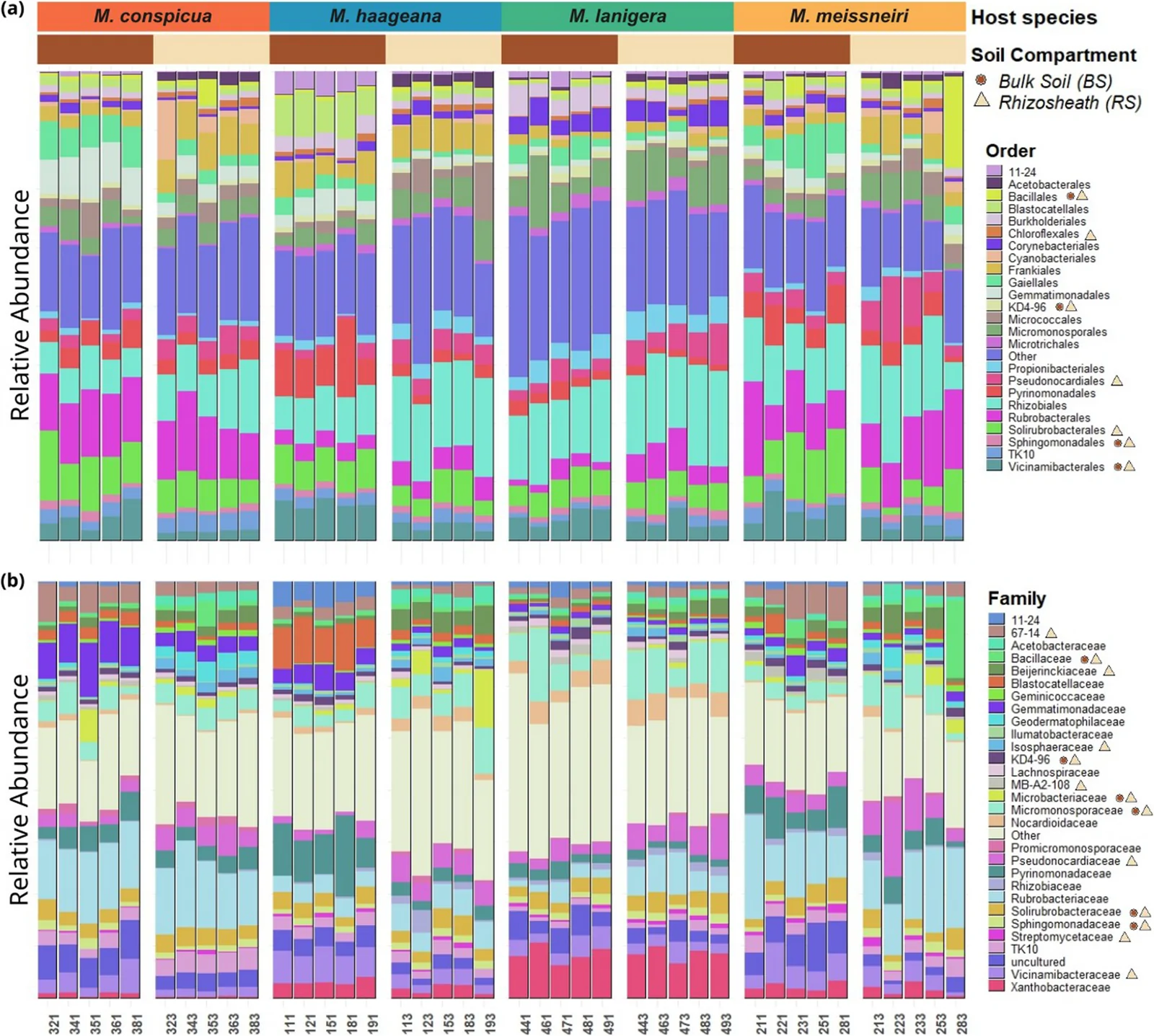
Contrasting bacterial taxonomic profiles across localities and compartments. Barplots show the relative abundance of the most representative bacterial orders (**a**) and families (**b**) associated with the MHSC. To improve readability, the number of taxa displayed in each panel was determined using the elbow point after ranking taxa by relative abundance (Supplementary Information Fig. S1). Numbers below each bar indicate sample IDs, corresponding to samples in Supplementary Information Table S1. Differences in taxon abundance were assessed using Kruskal-Wallis tests across localities within each soil compartment (BS and RS), followed by Holm correction (*p* ≤ 0.05). Taxa showing non-significant differences are indicated next to the taxon name using symbols: brown circles denote non-significant variation in BS samples, whereas wheat triangles denote non-significant variation in RS samples. Full statistical results are provided in Supplementary Information Table S2

At order level, the top 24 taxa (Fig. 2a) exhibited larger differences between BS than in RS. Bacillales, KD4-96, Sphingomonadales and Vicinamibacterales were dominant at all four sites and in both soil compartments. Orders Chloroflexales (H_(3)_ = 6.200, *p* = 0.102^n.s^.), Pseudonocardiales (H_(3)_ = 3.000, *p* = 0.392^n.s^.), and Solirubrobacterales (H_(3)_ = 1.697, *p* = 0.638^n.s,^) were dominant at the RS of the four MHSC species, while showing significant differences with their corresponding BS samples (H_(3)_ = 8.600, *p* = 0.035*, H_(3)_ = 8.074, *p* = 0.044*, and H_(3)_ = 10.748, *p* = 0.013*; respectively).

Similar patterns were observed for the top 29 families (Fig. 2b). Bacillaceae, KD-96, Microbacteriaceae, Micromonosporaceae, Solirubrobacteraceae, and Sphingomonadaceae dominated across both soil compartments and at all four locations. Several families were found in similar relative abundances at RS samples such as Beijenrickaceae, Isosphaeraceae, MB-A2-108, Pseudonocardiaceae, and Streptomycetaceae, among others. However, these same families differed significantly with their corresponding BS samples across all locations, suggesting positive recruitment for these taxa to favor their relative dominance in the RS (see Supplementary Information Table S2 for detailed statistics of the displayed taxa).

By analyzing dominant taxa across three taxonomic ranks (i.e. phylum, order, and family), we observed that most taxa exhibited significant differences on their relative abundances between location (BS samples) and between MHSC species (RS samples). This is expected given the geographical distance between the sampled locations and their corresponding contrasting environmental conditions. As differences in bacterial communities between locations were the main tendency, we highlighted the groups that show non-significant differences, which may be more informative to identify filtering patterns (Fig. 2).

First, we observed taxa showing non-significant differences among RS samples, but significant differences in BS. These taxa may be consistently enriched across all four MHSC species. In contrast, in the absence of the rhizosheath and its recruitment on the BS community, these taxa exhibit variable relative abundances likely influenced by local abiotic conditions. The absence of the opposite tendency (i.e. non-significant differences among BS samples but significant differences among RS samples) suggests that, among widely dominant soil bacteria, no differential preference between MHSC occurs when filtering them into the RS.

### Despite Differences Between Species and Soil Compartments, Similar Filtering Preferences are Shared by the MHSC Species

**Fig. 3:**
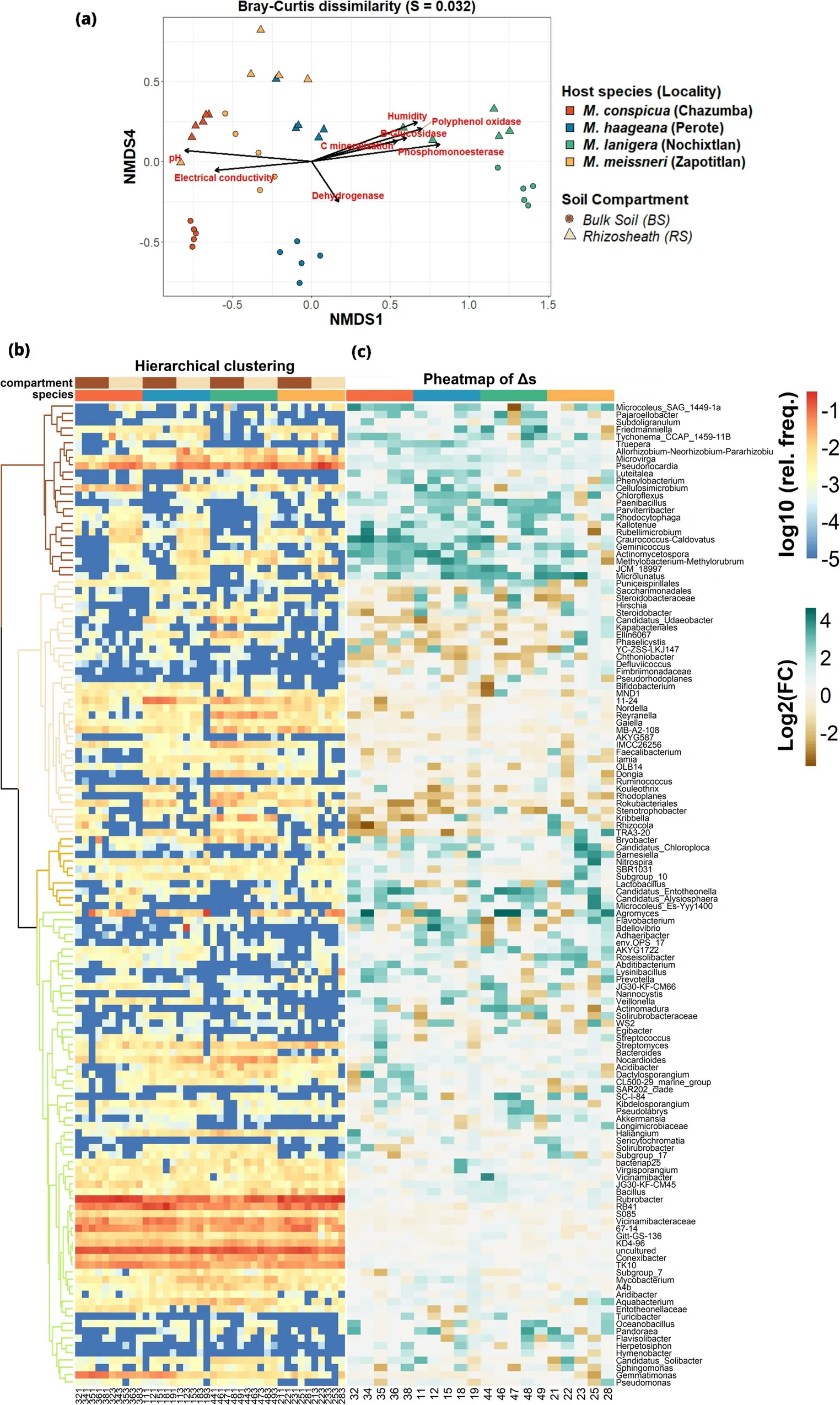
MHSC preferences influence the bacterial composition filtering in the rhizosheath. (**a**) NMDS ordination (S = 0.032) based on beta-diversity (Bray-Curtis dissimilarity): NMDS1 distinguishes between the four localities (colors), and axis NMDS4 shows the difference between compartments; vectors represent significantly (*p* ≤ 0.01) correlated biogeochemical processes and soil properties. (**b**) Log_10_ relative abundance of the top 134 genera among localities and compartments with sample IDs specified at the bottom. (**c**) Relative enrichment (Δ) in the RS compared to BS shown as Log_2_ (Fold Change), with paired sample IDs specified at the bottom (i.e., “32” is the division of the RS sample “323” over the BS sample “321”). Both heatmaps (**b**, **c**) are assorted by similarity according to a hierarchical clustering (Ward algorithm) on the Fold Change values. Dendrogram colors represent the main clusters

To explore overall patterns across RS and BS samples, we performed multivariate analyses using bacterial genus-level data to increase taxonomical resolution. Beta diversity was estimated using Bray-Curtis dissimilarity and visualized through non-metric multidimensional scaling (NMDS, Fig. 3a), with a low stress value (0.032), indicating a reliable ordination of the data. As all NMDS axes are equally explicative, we chose the pair that better highlighted the differences between bacterial communities (Supplementary Information Fig. S2). Correlation of NMDS axes with soil biogeochemical variables using vegan::envfit() revealed that NMDS1 was associated with differences among BS samples from each location (r^2^ = 0.7521, *p* = 0.0001***), primarily driven by variation in enzymatic activity related to carbon (C) and P cycling, and soil pH. In contrast, axis NMDS4 better captured the variation associated with soil compartment, distinguishing BS from RS samples (r^2^ = 0.0969, *p* = 0.0062**), though no fitting could be conducted on the environmental vectors as they only represent BS soil biogeochemical and physical properties, but not RS properties. Overall, BS and RS samples were clearly separated in the ordination space, while host species-level differences were also evident. However, *M. haageana* and *M. meissneri* partially overlapped along NMDS1, indicating similar community composition under certain environmental conditions.

To further explore patterns of genus-level abundance among the localities and between soil compartments, we analyzed the top 134 genera, clustering them according to relative abundance (Fig. 3b), and calculating the differential between paired RS and BS samples (Δ values, Fig. 3c). Within each site, both compartments showed differential enrichment of dominant bacterial genera. Nevertheless, MHSC species displayed similar enrichment patterns in their RS relative to the surrounding BS, as reflected by consistent Δ values across sites. Some genera are overall highly abundant at the four localities and both compartments, such as *Gaiella*, *Gemmatimonas* or *Nordella*; yet these are not noticeably enriched in the RS or can even be mildly depleted from the RS. Contrarily, genera such as *Agromyces*, *Craurococcus-Caldovatus* and *Microlunatus* are enriched in the RS, making them candidate groups for recruitment by MHSC species. Genera such as *Microvirga*, *Pseudonocardia*, and *Rubrobacter*, exhibit slight enrichment in the RS relative to the BS; however, their overall high relative abundance renders them less sensible to fold change (FC). Meanwhile, genera such as ca. *67 − 14*, *Gemmatimonas* and *Vicinamibacter* were universally depleted from the RS.

### The Common Core in MHSC Species Comprises 17 Bacterial ASVs

**Fig. 4:**
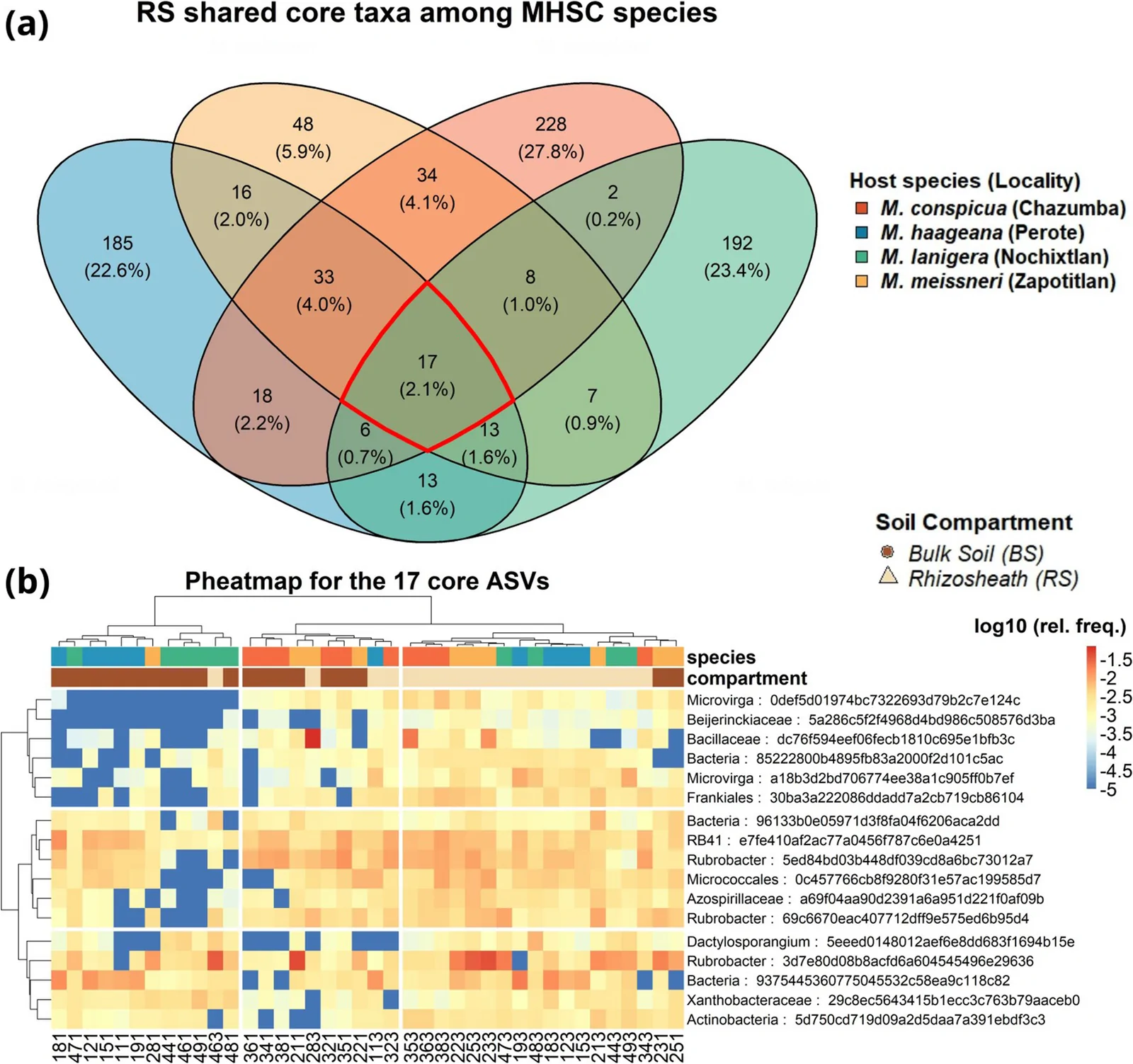
The taxonomic (or common) core found in the RS of MHSC. (**a**) RS shared core taxa among species (highlighted with a red line): Venn diagrams for enriched ASVs in the RS for all analyzed MHSC species. (**b**) Heatmap of 17 ASVs. On top of the heatmap, two rows of color indicate the species/locality (top row) and the compartment (bottom row). Rows (3 main clusters) and columns (3 main clusters) order are based on a hierarchical clustering using Ward algorithm, while breaks highlight main clusters identified using the silhouette method. Sequencing sample IDs are specified at the bottom

To identify ASVs defining the BCM, we applied the ‘common core’ concept [4]. ASVs were considered part of the common BCM if they met the following criteria: (i) the relative abundance for any given ASV must be higher in the RS than in BS samples; (ii) ASVs must occur in at least 4/5 RS samples per MHSC species; and (iii) any given ASVs must also have a minimum relative abundance of 0.001 in all RS samples where it occurs. 1,382 ASVs in the RS met these criteria, but only 17 ASVs were shared by all four sampled MHSC species (Fig. 4a). These 17 ASVs constitute the common BCM and together they represent a median relative abundance of 6.22% within RS of the *Mammillaria* species. Of these ASVs, some could be taxonomically assigned to the genus level, including *Microvirga* and *Rubrobacter* (enriched in the RS), *Dactylosporangium* (showing mixed enrichment/depletion in the RS) and the uncultured genus *ca. RB41* (slightly enriched in the RS). Additionally, conducting this analysis at genus level (minimum relative abundance > 0.005) revealed a set of 13 genera closely associated with the MHSC species, including known plant growth promoting bacteria such as *Agromyces*, *Conexibacter* and the arid-thriving *Pseudonocardia* (Supplementary Information Fig. S3a).

To confirm their relevance for defining the BCM, we performed hierarchical clustering across all samples (Fig. 4b), retrieving three major groups. The first group (the “Sub-humid cluster”) grouped together all BS samples from Perote and Nochixtlan — our most mesic localities —, and only a single RS sample from *M. lanigera*. The second group (the “Arid cluster”) consisted predominantly of BS samples from Chazumba, along with two BS samples from Zapotitlán and three RS samples — each of these RS samples coming from one of the three species *M. conspicua*, *M. haageana*, and *M. meissneri* respectively. The third cluster recovered nearly all of the remaining RS samples, along with two of the five BS samples from Zapotitlán, the driest locality and the one inhabited by *M. meissneri*. This ordination confirms that the relative abundances of these ASVs are strongly diagnostic of the RS microbiome, and appear more closely linked to the RS compartment than to the local BS community.

In contrast, the same analysis at genus level revealed that BS samples differed from their corresponding RS samples in their enrichment at these 13 core genera, except for the RS samples of *M. conspicua*, which were clustered with the BS samples. This suggests that the filtering exerted by the rhizosheath of *M. conspicua* is less deterministic at genus-level but more specific at a ASV level (Supplementary Information Fig. S3b). Furthermore, the clustering by sample and genus allowed us to visualize that, even when none of the 17 core ASVs were members of genus *Agromyces*, this genus is highly enriched in the RS samples but barely present in the BS. This could indicate a higher-level filtering by the MHSC recruiting closely related bacteria instead of a particular population-like entity.

Row clustering also produced three main groups. The upper cluster contained ASVs that were consistently rare in BS but strongly enriched in the RS across species. The middle cluster included ASVs that tended to be rarer in samples from the “Sub-humid cluster” (i.e. Perote+Nochixtlan) than in those from the “Arid cluster” (i.e. Chazumba+Zapotitlán). Conversely, the lower cluster consisted of ASVs that were less abundant in the “Arid cluster” and comparatively more common in the “Sub-humid cluster.” Together, this structure indicates that the shared RS core is composed of bacteria broadly enriched in the RS across all four species, suggesting filtering processes that are conserved throughout the MHSC.

### Common Functional Predictions in the MHSC Complex

Once we identified the common core, we proceeded to identify the functional core. We used PICRUSt2 to identify common functional processes shared by the bacterial communities. We searched for the main metabolic pathways associated with each locality and compartment (Supplementary Information Fig. S4), predicted according to the MetaCyc database. Similar to our treatment of taxa (Fig. 1), we ranked the pathways from highest to lowest relative frequency and analysed the top fraction as calculated by the elbow point (top 37 pathways). These main pathways are associated with common bacterial cellular processes like cell-wall production (e.g. PW0-1586, PWY-5659, DTDPRHAMSYN-PWY), membrane maintenance (e.g. PHOSLIPSYN-PWY, PWY-6121, PWY66-389), amino acid biosynthesis (e.g. ILEUSYN-PWY, THRESYN-PWY, VALSYN-PWY), energy acquisition and nutrition (e.g. PWY-5130, PWY-5695, PWY-7111), and communication (PWY-7663). These results are expected as they relate to primary metabolism in bacterial communities.

Nevertheless, each locality showed some particularities. Bacterial communities from Perote BS are predicted to favor colanic biosynthesis (COLANSYN-PWY), which is involved in biofilm formation. There was also predicted enhanced taurin degradation (PWY-6969), which is linked to sulfur soil cycle, in BS and RS samples. In Chazumba there were more ASVs predicted to be capable of conducting responses against abiotic stress (PWY-5103, PWY-5686). Soils from Zapotitlan had higher anaerobic metabolic potential (PWY-7977). Finally, at Nochixtlan we observed a higher predicted abundance of pathways related to fatty acid oxidation (PWY-5686) and the citrate cycle (PWY-702); pathways associated with increased metabolic activity of the microbial community [36].

**Fig. 5:**
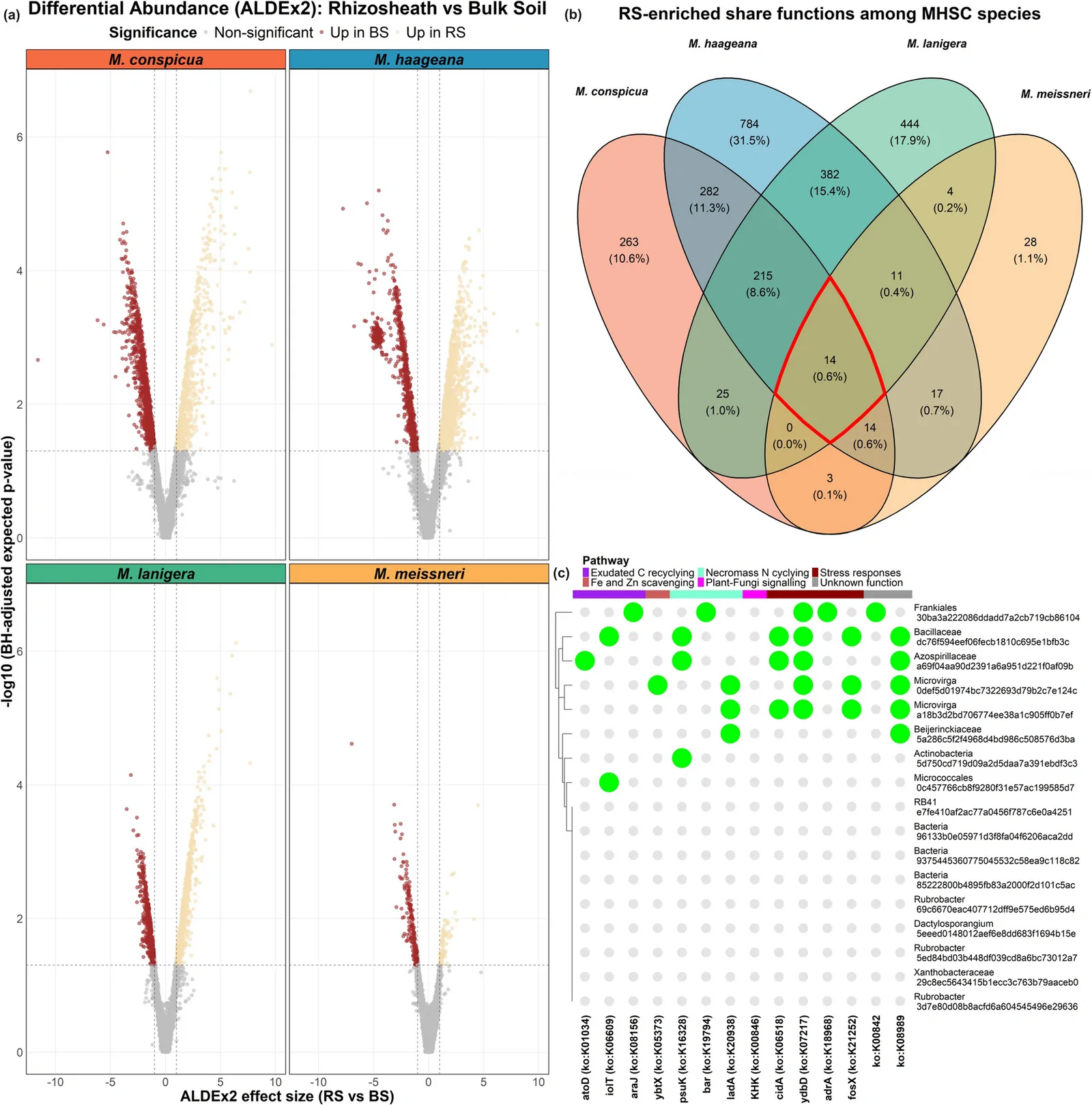
The predicted functional core bacteriome suggests enrichment of functional potentials related to plant growth. (**a**) Volcano plots based on species-specific independent ALDEX2 models for the KEGG Orthology (KOs) for functions predicted as enriched in the RS (wheat) or enriched in the BS (brown). Highlighted KOs have an absolute value larger than 10 for the log_2_ fold change and a significance level above 2 for the -log_10_ of adjusted p-value. (**b**) Venn Diagram of the main shared KOs that were differentially enriched in RS. (**c**) Binary heatmap displaying ASVs (rows) of the common core and their predicted KOs (columns) from the functional core. Rows are clustered according to Ward method, while columns are grouped in custom-made categories based on results reported in literature

In order to compare the functional profiles of both soil compartments and their behavior for each MHSC species, we conducted an ALDEX2 normalization, as recommended for PICRUSt2 output, to seek for finer biogeochemical processes using the predicted KEGG Orthology (KO) terms and visualized it in a volcano plot (Fig. 5a), which showed that each species has different predicted functional processes up- or down-predicted in the RS.

We then analysed KO terms and obtained 14 core KOs (Fig. 5b). 13 of these KOs are predicted to be present in the genome of at least one ASV from the common core definition (Fig. 4a, b), and are annotated as being associated with various functional processes such as recycling of root exudates (KO abb.: araJ, atoD, iolT), metal scavenging (KO abb.: ybtX), responses to biotic and abiotic stress (KO abb.: adrA, cidA, fosX, ydbD), acquisition of carbon (C) and Nitrogen (N) from soil necromass (KO abb.: bar, ladA, psuK), or some as yet unknown function (K00842, K08989). The only KO that was not associated with the common core was ketohexokinase (KO abb.: KHK), an enzyme related to root-exudated fructose degradation and indirectly linked to changes in bacterial activity via effects on hyphal metabolism [37].

Specifically, the ASVs assigned to *Microvirga* were predicted to encode enzymes related to Fe and Zn scavenging, necromass N acquisition and responses to biotic and abiotic stress; meanwhile uncultured members of the Frankiales, Bacillaceae and Azospirillaceae also were predicted to encode KOs associated with degradation of exudated C (Fig. 5c). On the other hand, genera such as *Dactylosporangium* or *Rubrobacter* were not predicted to encode these core KOs; this could suggest that these ASVs either conduct functional processes that are not captured in the data used by PICRUSt2 or, alternatively, that their importance relies on their ecological role within the rhizosheath bacteriome associated to the MHSC. This could be analyzed through co-occurrence networks or directly measuring the functional processes, but that is a perspective that lies outside the experimental design of this work.

Together, these results suggest that, driven by functional capabilities linked to a set of phylogenetically related ASVs, the MHSC has been able to colonize contrasting environments with varying degrees of aridity by putatively establishing a holobiotic association linked to the formation of an RS layer, which enables the whole root-bacteria system to survive varying degrees of environmental stress and recycle necromass nutrients.

## Discussion

Here, we provide the first comprehensive characterization of the common and functional bacterial core microbiome across four closely related species from the *Mammillaria haageana* species complex that have diverged into contrasting and geographically distant environments along an aridity gradient. Using abundance-based criteria and cross-species occurrence thresholds, we identified 17 ASVs that consistently distinguish the *Mammilaria* rhizosheath from surrounding bulk soil. Rhizosheath is predicted to be enriched in KO terms involved in C and N acquisition from the necromass, a biogeochemical process that dominates nutrient cycling under resource scarcity in arid soils [38]. The predicted rhizosheath functional potentials included pathways for biofilm formation and root responsiveness that may alleviate environmental stress. Altogether, these results suggest a close relationship between the BCM and the MHSC species that, despite environmental differences, has persisted in these closely related cacti species across their diversification (2 mya). Similar processes could have occurred during the steady radiation of Cactaceae in the Americas. Other works have demonstrated persistent associations with fungi that helped cacti colonize arid environments in recent allogenous dispersion events [39].

The four localities differed in soil biogeochemical activity (Fig. 3a), yet MHSC rhizosheaths share preferences for certain bacterial genera. One of the most enriched, *Microvirga*, includes two ASVs in the common BCM. Species in this genus have been characterized as nitrogen-fixers and especially enriched in alkaline and arid soils [40]. Experimentally, this genus has been isolated from roots of Fabaceae and also has been previously found enriched in Cactaceae roots of *Opuntia ficus-indica* from Tunisia [14]. These observations suggest that *Microvirga* could be important for N-recycling for plants from arid environments, as our KOs predictions indicate (barbiturase), yet no experimental work on *Microvirga* has been done to test this hypothesis, but the link between N-recycling in the rhizosphere and plant growth has been demonstrated elsewhere [38].

Two other enriched genera in the RS included *Rubrobacter* and *Pseudonocardia*. *Rubrobacter* contributed three ASVs to the common BCM and is a group described elsewhere in rhizospheres from xerophytes [41–42]. *Rubrobacter* has even been found as a signature of tolerance to metallic and hydric stress in the cactus *Echinocactus platyacanthus* [43]. Finally, *Agromyces* and *Pseudonocardia* were positively filtered by all four MHSC species and present in the core genera, though no particular ASV was identified as a member of the common BMC, maybe because they did not fulfill the minimum relative abundance (< 0.1%) or occupancy (⅘ RS samples) criteria. *Pseudonocardia* is interesting as this genus contains plant-growth promoting bacteria and is particularly abundant in desert soils and rhizospheres [13–14].

Furthermore, *Agromyces*, *Pseudonocardia* and *Rubrobacter* have been linked to auxin production [44], a phytohormone that promotes lateral root formation and increased root hair density, which could be related to promoting rhizosheath formation as they increase the contact surface between soil particles and root exudates. Assays focused on measuring the functionality associated to these genera may shed light on this hypothesis which holds important implications as lateral and adventitious root growth are traits in *Mammillaria* root architecture that enhance water uptake efficiency after rainfall in arid environments [45–46].

Nonetheless, no KO terms directly linked to auxin production were detected in the functional core. However, *iotL* (*myo-*inositol) has been shown to regulate calcium uptake in roots, thereby enhancing root growth and root hair development. Inositol metabolism has also been linked to regulating auxin concentrations via PIN proteins polarity [47]. Further analyses using direct techniques such as transcriptomics are needed to fully understand the relation of MHSC functional core responses to varying environmental conditions.

Based on the clustering for the common core (Fig. 4b) and the number of shared KOs enriched in the RS (Fig. 5b), we can recognize similar patterns for the “Sub-humid cluster” MHSC species, *M. haageana* and *M. lanigera*. Even though both species populations are hundreds of kilometers away, they resemble each other more than *M. conspicua* and *M. meissneri*, which are only a few kilometers apart. This pattern is consistent with the findings of Cervantes et al. [15], who suggested that the marked differences between *M. conspicua* and *M. meissneri* may be related with their distinct soil preferences. The analysis on core genera (Fig. S3) mostly captured the same pattern, though it suggests a weaker determinant effect by *M. conspicua* on the RS bacterial community as compared to the BS community, which may be interpreted as a more genus-generalists strategy rather than ASV-specific structure. Mentionworthy is that some genera like RB41 or TK10 were core and enriched in the RS despite their high abundance on the BS (Fig. 3b-c, Fig. S3). Whether all of these patterns are unique for cacti or shared with other xerophytes (i.e. cacti-driven vs. environment-driven recruitment), needs to be tested; as previous findings suggest that rhizospheres share similar bacterial communities despite the identity of the host species [48].

It is important to point out that the high number of sequences unclassified at phyla level (8.58%) suggest that a considerable proportion of the bacterial diversity from arid environments remains unexplored. Though this data is not reported explicitly by most previous works conducted on Cactaceae, it has been described that the relative abundance of unclassified bacteria at higher taxonomic ranks tends to increase proportionally to aridity [49]. Alternatively, this may be due to a high number of sequences from our samples pertaining to groups that are poorly represented within the SILVA database or due to a conservative confidence threshold (0.8) leaving many reads without a taxonomical annotation.

The functional BCM predicts that MHSC-selected microbes could be involved in nutrient acquisition (N, Fe and Zn) from soils. Given that most MHSC species inhabit oligotrophic soils, where nutrient inputs are sporadic and organic matter turnover is slow, the functional capability of recycling nutrients from decaying biological matter (especially N) becomes a major limitation in plant biomass acquisition. Predicted enrichment of KOs associated with Fe and Zn scavenging may enhance plant physiological activity [50–51] and promote a higher density of shallow lateral roots and the inhibition of primary root growth [52], which is characteristic of the MHSC complex. It is also noteworthy that the plant-fungi enzyme KHK was not predicted to be present in the genomes of the 17 core ASVs, but is predicted to be a highly enriched RS process across all four MHSC species. This is highly relevant as in *M. haageana* and *M. lanigera*, an endophytic fungus was constantly isolated during root architecture experiments [53], highlighting that in order to understand the MHSC microbiome we must look for the eukaryotic components of the community in the future.

Overall, our findings reveal that the MHSC shares a set of preferences that allow their rhizosheath to be colonized by a common BCM. This occurs even though soil communities differ in their enzymatic activity and taxonomic profiles. The shared BCM is in turn defined by a set of ASVs potentially related to growth-promoting and stress-alleviating bacteria, and functions such as N-acquisition from the necromass, Fe and Zn scavenging, and promotion of both, root and rhizosheath architecture development via root exudation and microbial biofilm growth promotion. Further exploration on the possible co-evolution of these bacteria with the MHSC must be specifically addressed to solve if close symbiotic relations may be hidden drivers of cacti radiations into arid zones. Our results advance the understanding of cactus–microbe interactions and provide a foundation for future work on the ecological roles and experimental validation of functional processes for rhizosheath-associated bacteria in cacti evolution. We need to explore interesting taxa such as *Microvirga*, *Pseudonocardia* and *Rubrobacter* that can be isolated and cultured in lab, making them potential candidates for a cacti-specific plant bacteria growth promoting (PBGP) inoculum, that could help in Cactaceae preservation and ecological restoration, as is the case of *Mammillaria san-angelensis*, a member of the MHSC which is facing extinction in the wild [54].

## Supplementary Information

Below is the link to the electronic supplementary material.

Supplementary Material 1

Supplementary Material 2

Supplementary Material 3

Supplementary Material 4

Supplementary Material 5

Supplementary Material 6

## Data Availability

The authors declare that the data supporting the findings of this study are available within the paper and its Supplementary Information files. Demultiplexed raw data (FASTQ) files are available on NCBI’s SRA database (BioProject: PRJNA1458857, accession numbers SRR38308263 to SRR38308303). Should any raw data files be needed in another format they are available from the corresponding author upon reasonable request.
